# Overexpression of miR-506-3p reversed doxorubicin resistance in drug-resistant osteosarcoma cells

**DOI:** 10.3389/fphar.2024.1303732

**Published:** 2024-02-14

**Authors:** Xinru Wang, Rumeng Ding, Zhe Fu, Meng Yang, Duolu Li, Yubing Zhou, Chongzhen Qin, Wenda Zhang, Liuzhe Si, Jingmin Zhang, Yuna Chai

**Affiliations:** ^1^ Department of Pharmacy, The First Affiliated Hospital of Zhengzhou University, Zhengzhou, Henan, China; ^2^ Department of General Surgery, The Third Affiliated Hospital of Zhengzhou University, Zhengzhou, Henan, China

**Keywords:** osteosarcoma, doxorubicin resistance, miR-506-3p, JAK2/STAT3 signaling pathway, U-2OS/Dox

## Abstract

**Background and objective:** Osteosarcoma is a common primary malignant tumor of bone, and doxorubicin is one of the most widely used therapeutic drugs. While the problem of doxorubicin resistance limits the long-term treatment benefits in osteosarcoma patients. The role of miRNAs and their target genes in osteosarcoma have become increasingly prominent. Currently, there is no report on miR-506-3p reversing doxorubicin resistance by targeting STAT3 in osteosarcoma. The purpose of this study was to investigate the molecular mechanism that overexpression of miR-506-3p reverses doxorubicin resistance in drug-resistant osteosarcoma cells.

**Methods:** Doxorubicin-resistant osteosarcoma cells (U-2OS/Dox) were constructed by intermittent stepwise increasing stoichiometry. The target genes of miR-506-3p were predicted by bioinformatics approach and the targeting relationship between miR-506-3p and STAT3 was detected using dual luciferase reporter assay. U-2OS/Dox cells were treated with miR-506-3p overexpression and STAT3 silencing respectively. Then Western blot and RT-qPCR were used to detect the protein and mRNA expression levels of JAK2/STAT3 signaling pathway, drug-resistant and apoptotic associated molecules. The migration and invasion were assessed by cell scratch assay and transwell assay. The cell proliferative viability and apoptosis were investigated by CCK8 assay and flow cytometry assay.

**Results:** U-2OS/Dox cells were successfully constructed with a 14.4-fold resistance. MiR-506-3p is directly bound to the 3′-UTR of STAT3 mRNA. Compared with U-2OS cells, the mRNA expression of miR-506-3p was reduced in U-2OS/Dox cells. Overexpression of miR-506-3p decreased the mRNA expression levels of JAK2, STAT3, MDR1/ABCB1, MRP1/ABCC1, Survivin and Bcl-2, and decreased the protein expression levels of p-JAK2, STAT3, MDR1/ABCB1, MRP1/ABCC1, Survivin and Bcl-2, and conversely increased Bax expression. It also inhibited the proliferation, migration and invasion of U-2OS/Dox cells and promoted cells apoptosis. The results of STAT3 silencing experiments in the above indicators were consistent with that of miR-506-3p overexpression.

**Conclusion:** Overexpression of miR-506-3p could inhibit the JAK2/STAT3 pathway and the malignant biological behaviors, then further reverse doxorubicin resistance in drug-resistant osteosarcoma cells. The study reported a new molecular mechanism for reversing the resistance of osteosarcoma to doxorubicin chemotherapy and provided theoretical support for solving the clinical problems of doxorubicin resistance in osteosarcoma.

## 1 Introduction

As a highly aggressive bone malignancy, osteosarcoma was the most common primary tumor originating from mesenchymal cells, with a high prevalence in adolescents aged 10–14 years and older adults over the age of 60 years (Fu et al., 2023). Currently, the first-line treatment for osteosarcoma includes surgery, radiotherapy and chemotherapy. Although the 5-year survival rate has improved, the treatment for advanced and recurrent patients is still unsatisfactory. Osteosarcoma only responds to high doses of chemotherapeutic agents and is highly susceptible to chemoresistance, which are the main reasons for poor prognosis in osteosarcoma patients. Moreover, for osteosarcoma patients who develop chemotherapy resistance, the overall 5-year survival rate was significantly reduced to less than 20% (Zhu et al., 2019). Chemotherapy resistance was detrimental to subsequent treatment plans for patients, and was one of the difficulties in treatment for the orthopedic community. Therefore, it is urgent to solve the problem of chemoresistance in osteosarcoma patients.

During the treatment for osteosarcoma, chemoresistance is the most important pathophysiological basis for malignant tumor proliferation, susceptibility to recurrence and metastasis. The mechanism of chemoresistance in osteosarcoma has not been fully elucidated. Possible mechanisms included alteration of the activity of DNA topoisomerase, increase in the activity of glutathione transferase, dysfunction of membrane transport, activation of autophagy, and enhancement of DNA damage repair, etc (Lilienthal and Herold, 2020). Currently, doxorubicin, also known as adriamycin, is the cornerstone of osteosarcoma treatment, and had a response rate of up to 40% on osteosarcoma (Chou and Gorlick, 2006). However, long-term use of doxorubicin can lead to drug resistance. The occurrence of resistance could be caused by a variety of factors, and relevant pathogenesis is mainly concentrated in the key membrane transport proteins including P-gP, MDR1/ABCB1, and MRP1/ABCC1 (Elfadadny et al., 2021).

As a confluence of many oncogenic signaling pathways, the activation of signal transducer and activator of transcription 3 (STAT3) could induce aberrant expression of genes associated with tumor cell proliferation, differentiation, apoptosis, and chemo-resistance, and thus promoted tumor transformation, causing poor prognosis in patients (Barre et al., 2007; Hu et al., 2019). JAK2/STAT3 is a classical signaling pathway in tumor research. Under normal physiological conditions, transient activation of JAK2/STAT3 is strictly regulated. However, sustained activation of JAK2/STAT3 signaling had been found in many solid tumors, such as breast cancer, which promoted solid tumorigenesis, tumor growth, angiogenesis, host immune evasion, apoptosis resistance, carcinogenesis, and metastasis (Yu et al., 2009; Mengie Ayele et al., 2022; Rah et al., 2022). It had been demonstrated that the inhibition of JAK2/STAT3 signaling pathway could decrease cell viability, invasion and migration of osteosarcoma cells and induced apoptosis in cancer cells (Jia et al., 2022; Li and Liu, 2023; Ma et al., 2023). In addition, doxorubicin resistance could be antagonized by blocking the activation of the JAK2/STAT3 pathway induced by IL-6 secretion (Lu et al., 2021). However, the profound mechanism of reversing doxorubicin resistance mediated by the STAT3 remains to be further discovered.

In recent years, great progress had been made in the study of tumor phenotype changes caused by the interaction of miRNA and target genes. Some studies had reported that miR-506 could inhibit the biological activity of glioma cells (Peng et al., 2016), human hepatocellular carcinoma cells (Su et al., 2019) and colorectal cancer cells (Wei et al., 2019) by targeting STAT3. MiR-506 was lowly expressed in osteosarcoma tissues, and upregulation of miR-506 level could inhibit osteosarcoma cell proliferation and invasion, and promoted osteosarcoma cell apoptosis (Yao et al., 2016; Hu et al., 2017; Jiashi et al., 2018; Li et al., 2021). So far, the role of miR-506-3p reversing doxorubicin resistance by regulating STAT3 in osteosarcoma has not been reported. In this study, we focused on investigating the mechanisms of miR-506-3p reversing doxorubicin resistance in osteosarcoma, and provided theoretical support for solving the clinical problems of doxorubicin resistance in osteosarcoma.

## 2 Results

### 2.1 Construction of drug-resistant osteosarcoma cell line

**FIGURE 1:**
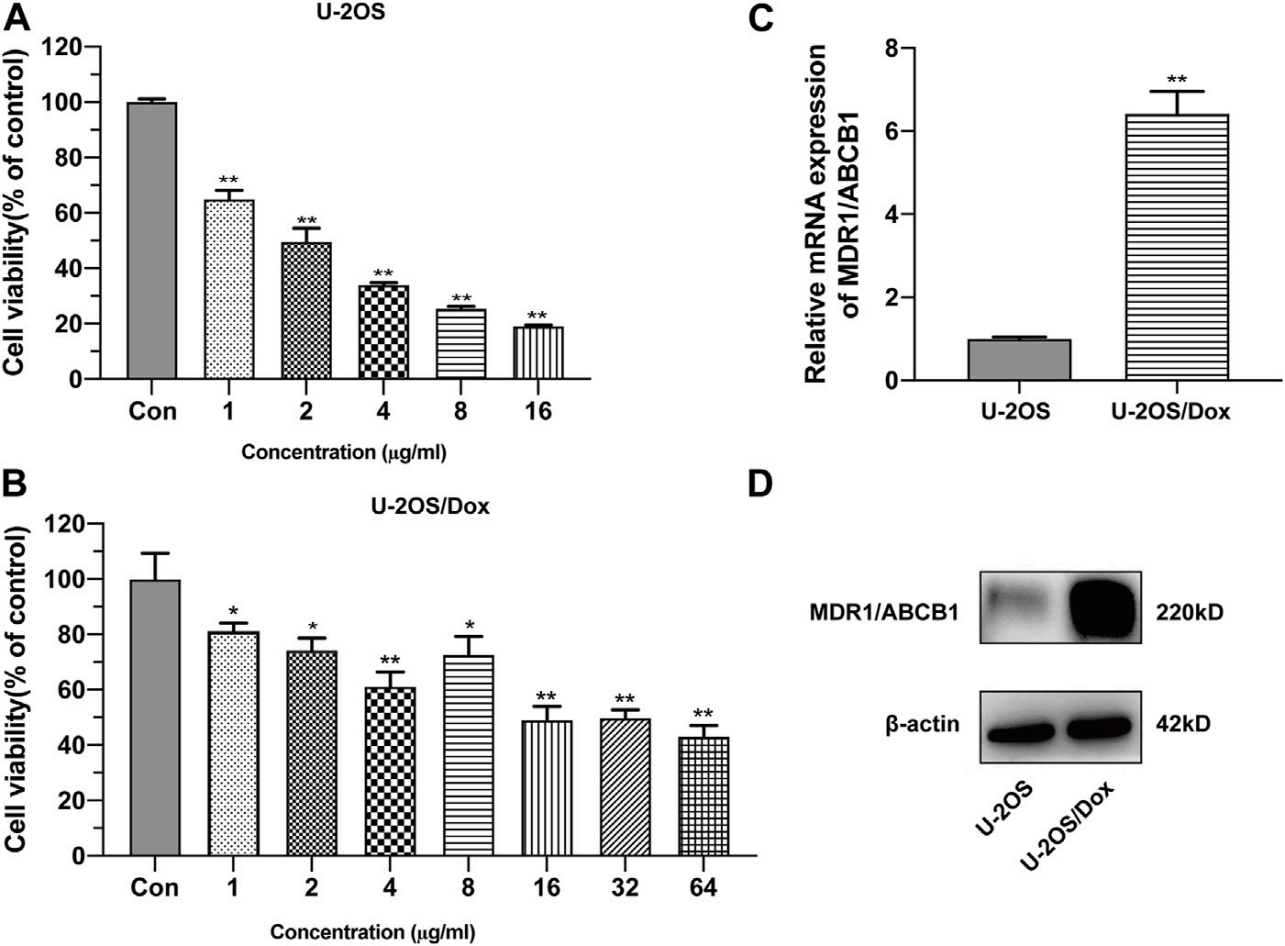
Construction of human osteosarcoma doxorubicin-resistant cell line U-2OS/Dox. The cell viability of drug-sensitive osteosarcoma cells U-2OS **(A)** and drug-resistant osteosarcoma cells U-2OS/Dox **(B)** were detected by CCK-8 assay with different concentrations of doxorubicin. IC_50_ values were detected in U-2OS (IC_50_ = 2.01 μg/mL) and in U-2OS/Dox (IC_50_ = 29.00 μg/mL) by CCK8 assay, RI = 14.4 fold. **(C, D)** The mRNA and protein expression levels of MDR1/ABCB1 in U-2OS and U-2OS/Dox were detected through RT-qPCR and Western blot, showing that MDR1/ABCB1 was significantly upregulated in U-2OS/Dox. *: *p* < 0.05, **: *p* < 0.01.

The half inhibitory concentration (IC_50_) values of drug-sensitive osteosarcoma cells (U-2OS) and doxorubicin-resistant human osteosarcoma cell line (U-2OS/Dox) were 2.01 μg/mL and 29.00 μg/mL, respectively. The resistance index (RI) was 14.4-fold (Figures 1A, B, *p* < 0.05). The mRNA and protein expression levels of MDR1 in U-2OS/Dox cells were significantly upregulated (Figures 1C, D, *p* < 0.01). The results from cell counting kit-8 (CCK8) and Western blot assays demonstrated that U-2OS/Dox was successfully constructed.

### 2.2 Results of bioinformatics analysis

#### 2.2.1 Target prediction of miR-506-3p

**FIGURE 2:**
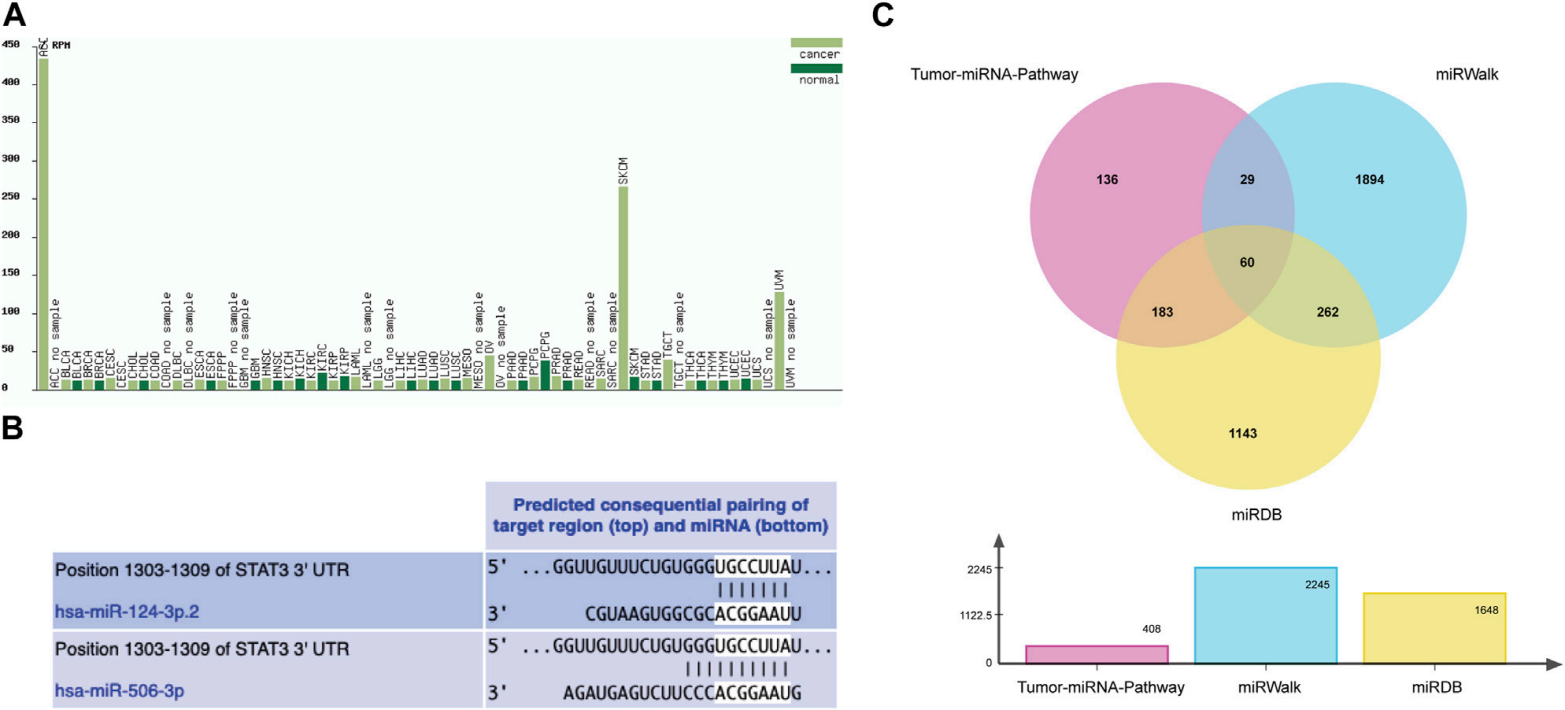
Tumor expression profile of miR-506-3p and prediction of binding sites and target mRNAs **(A)** Expression profiles of miR-506-3p in different tumor tissues and their paracancerous tissues, lacking the expression level of miR-506-3p in osteosarcoma. **(B)** The seed sequence of hsa-miR-506-3p was found to coincide with that of hsa-miR-124 by TargetScan Human 8.0 software prediction. **(C)** The Venn diagram of target genes prediction regulated by miR-506-3p in three databases.

The results of the cancer genome atlas (TCGA) database analysis showed that the change trends of miR-506-3p in different cancer types were not consistent, as shown in Figure 2A. However, the database lacks findings on the miR-506-3p in osteosarcoma, so this study explored the expression level of miR-506-3p in drug-sensitive and drug-resistant cells.

As predicted by TargetScan Human 8.0 database, the seed sequences of miR-506-3p and miR-124 tended to be consistent. Moreover, the binding site predicted by the 3′-UTR of miR-506-3p was broadly conserved with that of miR-124, as shown in Figure 2B.

From the Tumor-miRNA-Pathway database 408 genes were found, 1648 targets from the miRWalk database and 2245 genes from the miRDB database. Taking the intersection form by Wayne’s analysis finally yielded 60 genes, and STAT3 was one of the target genes that miR-506-3p might regulate, as shown in Figure 2C.

#### 2.2.2 GO and KEGG enrichment analysis of miR-506-3p target genes

**FIGURE 3:**
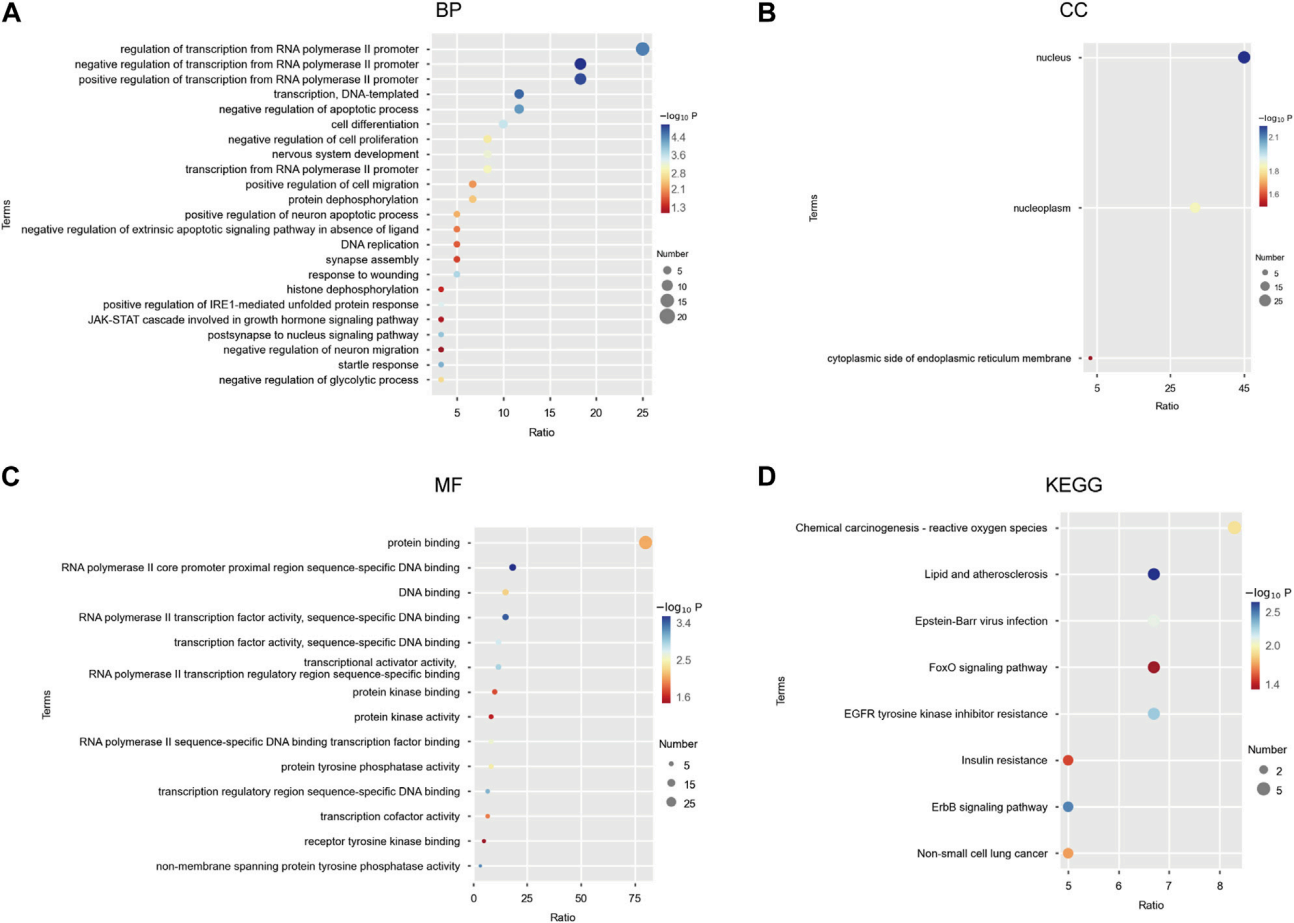
GO functional and KEGG pathway enrichment analysis of miR-506-3p target mRNAs **(A)** Twenty-three biological processes (BP) associated with miR-506-3p target genes and gene products by GO enrichment analysis; **(B)** Three cellular components (CC) associated with miR-506-3p target genes by GO enrichment analysis; **(C)** Fourteen molecular functions (MF) associated with miR-506-3p target genes by GO enrichment analysis. **(D)** Eight important signaling pathways associated with miR-506-3p target genes by KEGG enrichment analysis.

We performed Gene Ontology (GO) and Kyoto Encyclopedia of Genomes (KEGG) pathway enrichment analysis of miR-506-3p predicted target genes. Among them, GO enrichment analysis was used to characterize the relevant biological processes (BP), cellular components (CC) and molecular functions (MF). We observed that these target genes significantly clustered 23 terms in BP, 3 terms in CC, and 14 terms in MF (Figures 3A–C). They might be involved in apoptosis, cell proliferation and cell migration, which were mediated by transcriptional activator or tyrosine kinase signaling pathways, including the JAK-STAT. KEGG enrichment analysis identified eight important signaling pathways (Figure 3D), including the chemical carcinogenesis-reactive oxygen species signaling pathway, the Forkhead Box O (FoxO) signaling pathway, the epidermal growth factor receptor-tyrosine kinase inhibitor (EGFR-TKI) drug resistance signaling pathway, and the receptor protein-tyrosine kinase (ErbB) signaling pathway, etc.

#### 2.2.3 Protein-protein interaction (PPI) networks of miR-506-3p target genes

**FIGURE 4:**
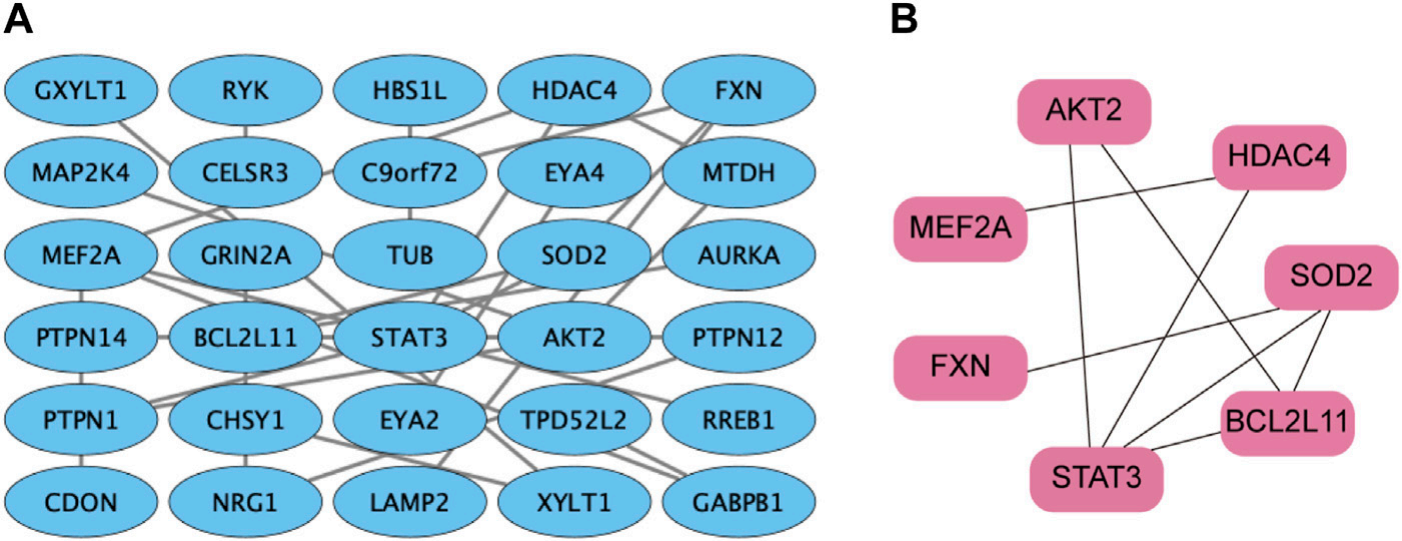
PPI network of miR-506-3p target genes **(A)** PPI network of miR-506-3p target genes were constructed by the STRING database. **(B)** The key genes targeted by miR-506-3p, including seven pivotal target genes, namely, STAT3, AKT2, BCL2L11, MEF2A, SOD2, FXN and HDAC4 were screened out by the cytoHubba plug-in and degree topology algorithm in Cytoscape software (version 3.8.0).

The STRING database was utilized to screen the target genes of miR-506-3p. PPI networks were established (Figure 4A), and then, were imported into Cytoscape software. The key genes targeted by miR-506-3p were acquired through the cytoHubba plug-in, including STAT3, RAC-beta serine/threonine-protein kinase (AKT2), B-cell lymphoma 2-like 11 (BCL2L11), Myocyte enhancer factor 2A (MEF2A), Recombinant superoxide dismutase 2 (SOD2), Frataxin (FXN), and Histone deacetylase 4 (HDAC4) (Figure 4B), which are hub target genes that may be involved in regulating osteosarcoma development and the molecular mechanisms of chemoresistance. Our previous studies found that the inhibition of STAT3 expression could reverse the chemosensitivity of osteosarcoma cells to doxorubicin, the STAT3 gene was chosen as a candidate target of miR-506-3p for the study of the molecular mechanism of reversing doxorubicin chemoresistance in drug-resistant osteosarcoma cells.

### 2.3 MiR-506-3p suppressed STAT3 expression by directly targeting the STAT3 3′-UTR

**FIGURE 5:**
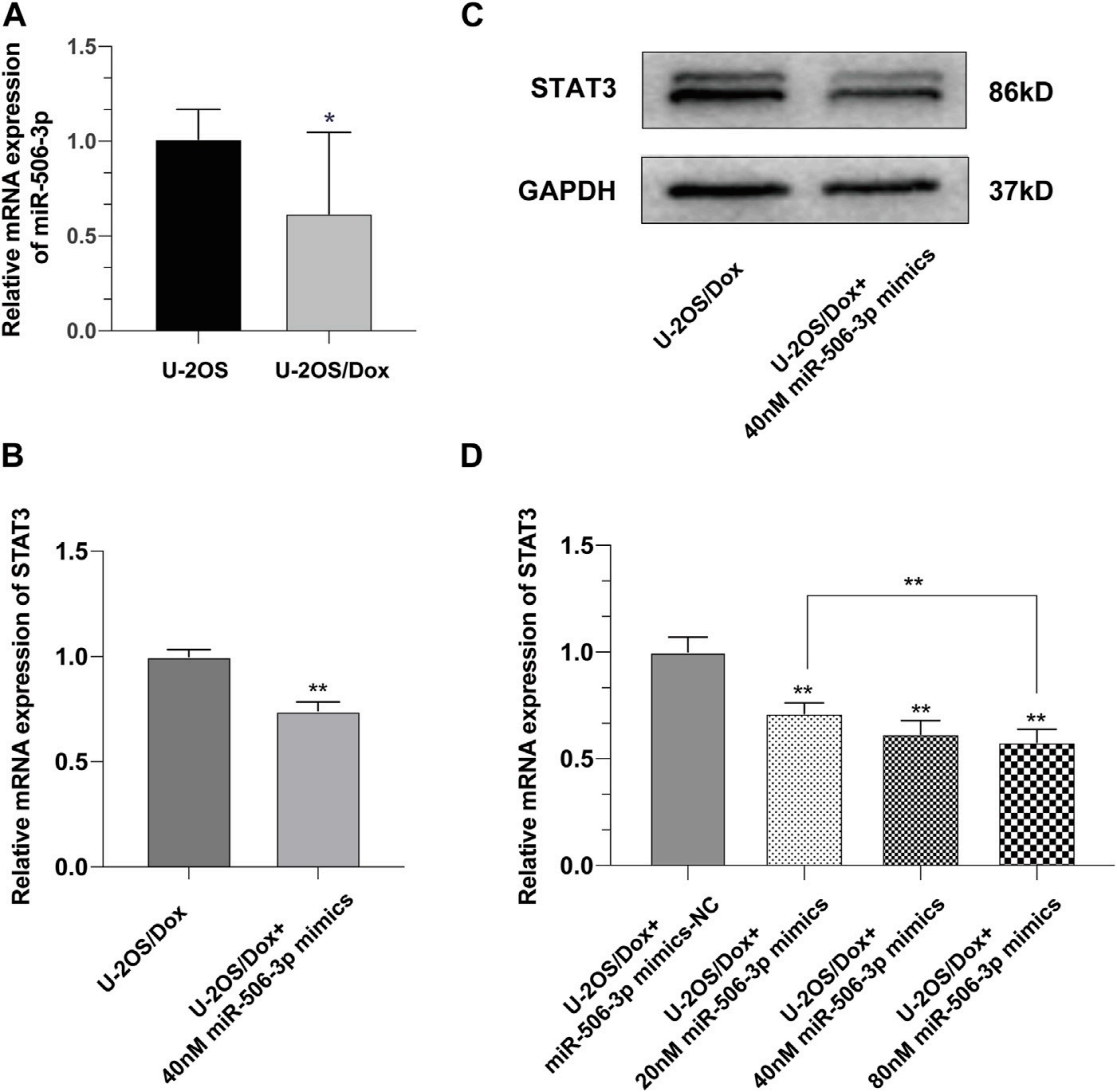
Overexpression of miR-506-3p downregulated the expression of STAT3 in U-2OS/Dox **(A)** The expression level of miR-506-3p in osteosarcoma-resistant cell line U-2OS/Dox was significantly lower than that in its parental cell line U-2OS. **(B, C)** The mRNA and protein expression levels of STAT3 were downregulated after transfection of 40 nM miR-506-3p mimics in U-2OS/Dox cells. **(D)** The inhibitory effect of miR-506-3p mimics on STAT3 was enhanced with the increasing concentration of miR-506-3p mimics. *: *p* < 0.05, **: *p* < 0.01.

Real time quantitative polymerase chain reaction (RT-qPCR) results showed that the expression level of miR-506-3p in U-2OS/Dox was significantly lower than that in its parental cell line U-2OS (Figure 5A, *p* < 0.05). After transfection of U-2OS/Dox cells with miR-506-3p mimics, we found that transfection of 40 nM miR-506-3p mimics downregulated STAT3 expression at the mRNA and protein levels (Figures 5B, C, *p* < 0.01). With the increasing concentration of miR-506-3p mimics, its inhibitory effect on STAT3 was also gradually enhanced (Figure 5D, *p* < 0.01). It indicated that miR-506-3p was negatively correlated with STAT3 expression in U-2OS/Dox cells relative to U-2OS, and overexpression of miR-506-3p inhibited STAT3 expression in U-2OS/Dox cells.

**FIGURE 6:**
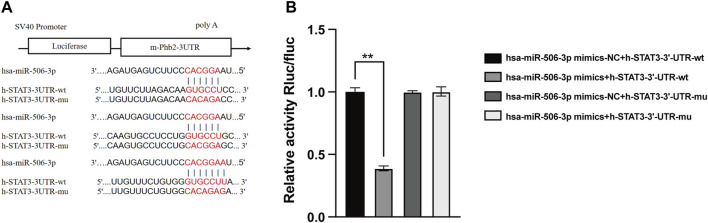
MiR-506-3p targeting STAT3 by dual luciferase reporter gene assay **(A)** Prediction of potential binding sites of miR-506-3p to the 3′-UTR of STAT3. **(B)** Dual luciferase reporter gene assay showed a significant decrease in the relative activity of STAT3 wild-type luciferase-expressing vectors. **: *p* < 0.01.

As verified by the dual luciferase reporter gene assay, the relative activity of STAT3 wild-type luciferase expression vector was significantly decreased to 0.291 ± 0.014 after transfection with miR-506-3p mimics, whereas that of the other three controls was 0.748 ± 0.025, 0.743 ± 0.007 and 0.746 ± 0.028 (Figure 6, *p* < 0.01). It could be seen that miR-506-3p bound directly to STAT3 3′-untranslated region (3′-UTR), indicating that there may be a targeted regulatory relationship between miR-506-3p and STAT3.

### 2.4 Effects of miR-506-3p overexpression and STAT3 silencing on migration and invasion of U-2OS/Dox cells

**FIGURE 7:**
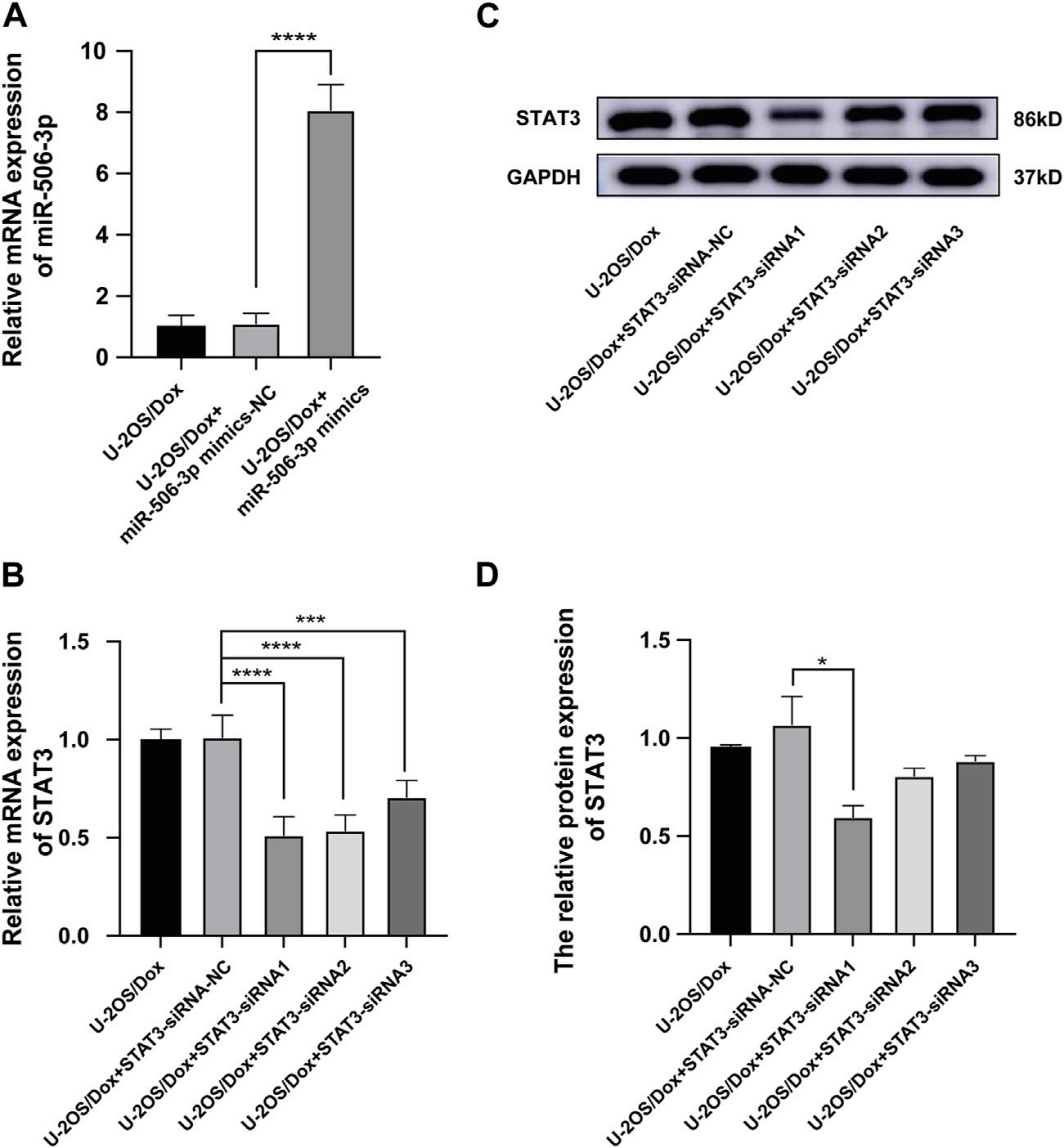
The Screening of STAT3 siRNAs **(A)** The miR-506-3p expression level in U-2OS/Dox + miR-506-3p mimics group was significantly higher than that in the U-2OS/Dox control group or U-2OS/Dox + miR-506-3p mimics-NC group by RT-qPCR detection, indicating that transfection was successful. **(B–D)** STAT3-siRNA1 had the best silencing effect on STAT3 gene by RT-qPCR and Western blot experiments. *: *p* < 0.05, **: *p* < 0.01.

MiR-506-3p mimics and mimics-NC were transfected into U-2OS/Dox cells using liposome transfection technology. RT-qPCR assay of the transfection efficiency showed that the miR-506-3p expression level in the mimics group was significantly higher than that in the control and mimics-NC groups, indicating successful transfection (Figure 7A, *p* < 0.01). The gene silencing technology was utilized to cause low expression of STAT3 in U-2OS/Dox cells. RT-qPCR and Western blot experiments showed that the expression level of STAT3 in the STAT3-siRNA1 group was significantly reduced compared with that in the STAT3-siRNA-NC group, STAT3-siRNA2 and STAT3-siRNA3 (Figures 7B–D, *p* < 0.05). Thereby, STAT3-siRNA1 had the best effect on gene silencing among all the groups tested and was chosen for the following experiments.

**FIGURE 8:**
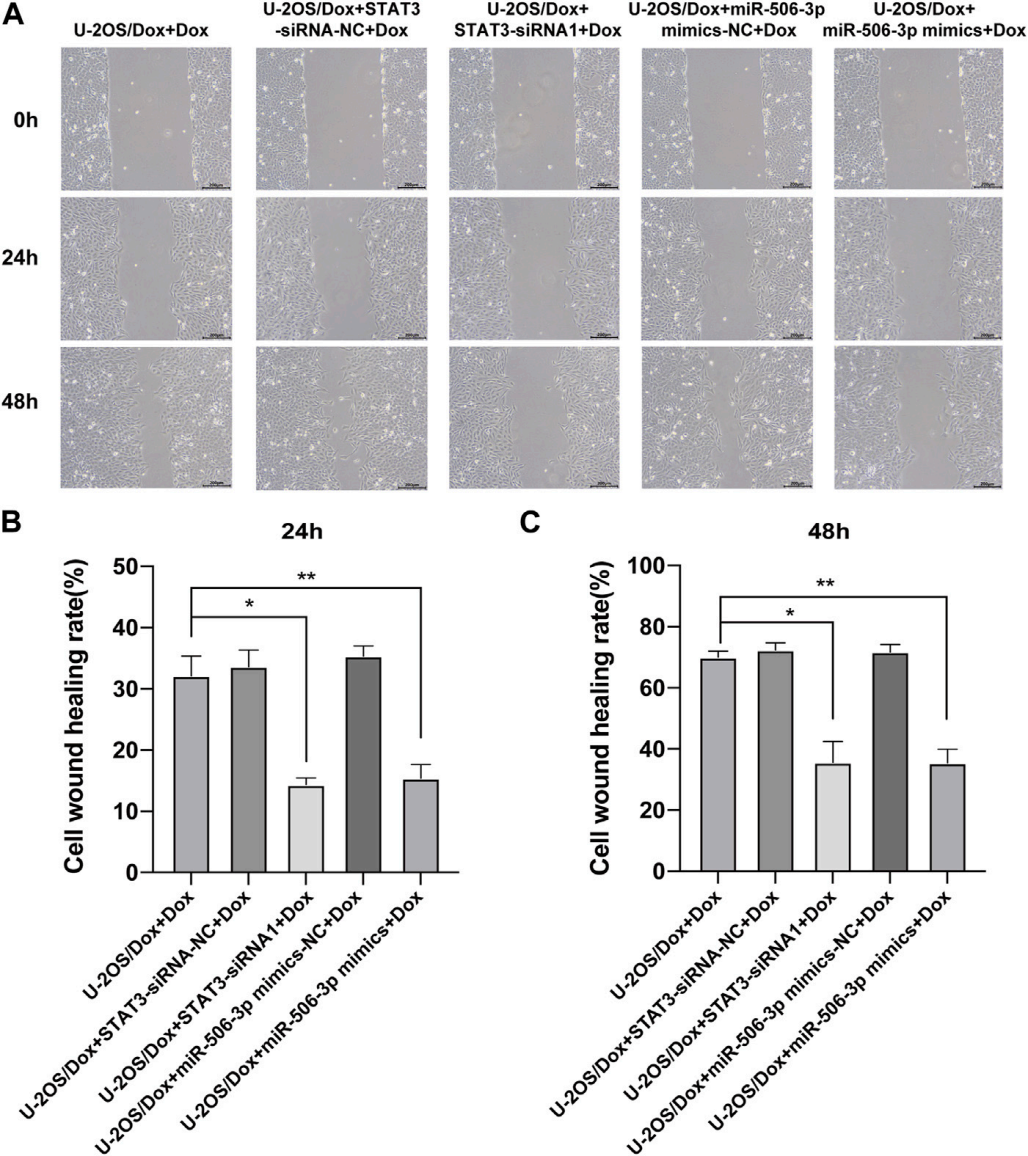
The migratory abilities of drug-resistant osteosarcoma cells were both inhibited by miR-506-3p overexpression and silencing STAT3 **(A)** The cell healing images of scratches at 0 h, 24 h or 48 h by cell scratching assay (magnification, ×100). The number of migrated cells in the U-2OS/Dox were both significantly reduced in the overexpression of miR-506-3p group (U-2OS/Dox + miR-506-3p mimics) and the silencing of STAT3 group (U-2OS/Dox + STAT3-siRNA1) after 24 h **(B)** or 48 h **(C)** of co-culture, compared with the drug-resistant osteosarcoma cells spiked group (U-2OS/Dox + Dox). *: *p* < 0.05, **: *p* < 0.01.

**FIGURE 9:**
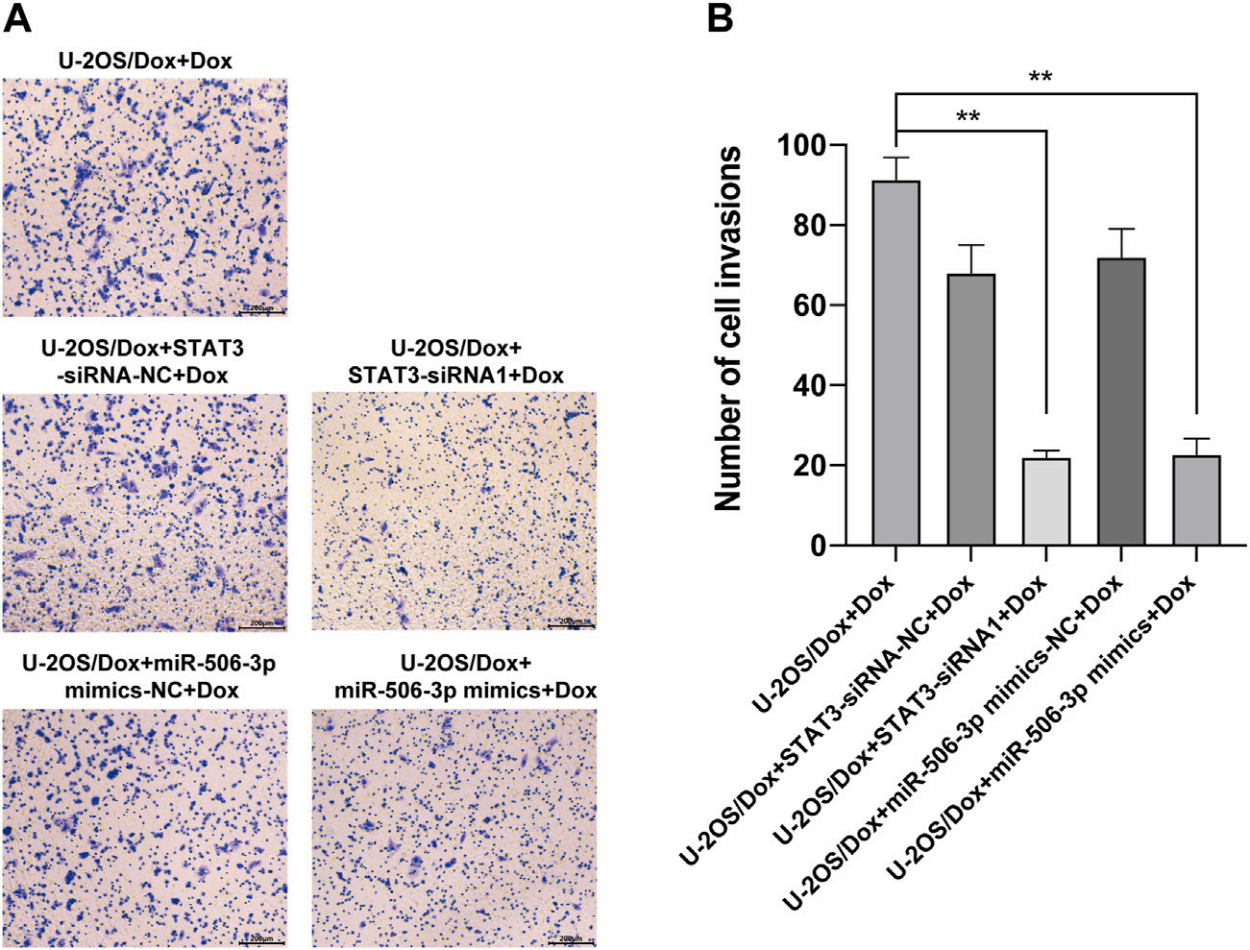
The invasive abilities of drug-resistant osteosarcoma cells were both inhibited by miR-506-3p overexpression and silencing STAT3 **(A)** The cell migration rates were detected by the transwell assay under an inverted microscope (magnification, ×100) after 24 h incubation. **(B)** The number of invasive cells in the U-2OS/Dox were both significantly reduced in the overexpression of miR-506-3p group (U-2OS/Dox + miR-506-3p mimics) and the silencing of STAT3 group (U-2OS/Dox + STAT3-siRNA1) after 24 h co-culture, compared with the drug-resistant osteosarcoma cells spiked group (U-2OS/Dox + Dox). *: *p* < 0.05.

The results showed that after doxorubicin was added to each group (U-2OS/Dox group, U-2OS/Dox + miR-506-3p mimics-NC group, U-2OS/Dox + miR-506-3p mimics group, U-2OS/Dox + STAT3-siRNA-NC group and U-2OS/Dox + STAT3-siRNA1 group), compared with the control group (U-2OS/Dox + Dox), the numbers of migrating cells (Figure 8, *p* < 0.05) and invading cells (Figure 9, *p* < 0.05) were significantly reduced in the miR-506-3p overexpressing group (U-2OS/Dox + miR-506-3p mimics) and STAT3 silencing group (U-2OS/Dox + STAT3-siRNA1).

### 2.5 Effects of miR-506-3p overexpression and STAT3 silencing on proliferation and apoptosis of U-2OS/Dox cells

**FIGURE 10:**
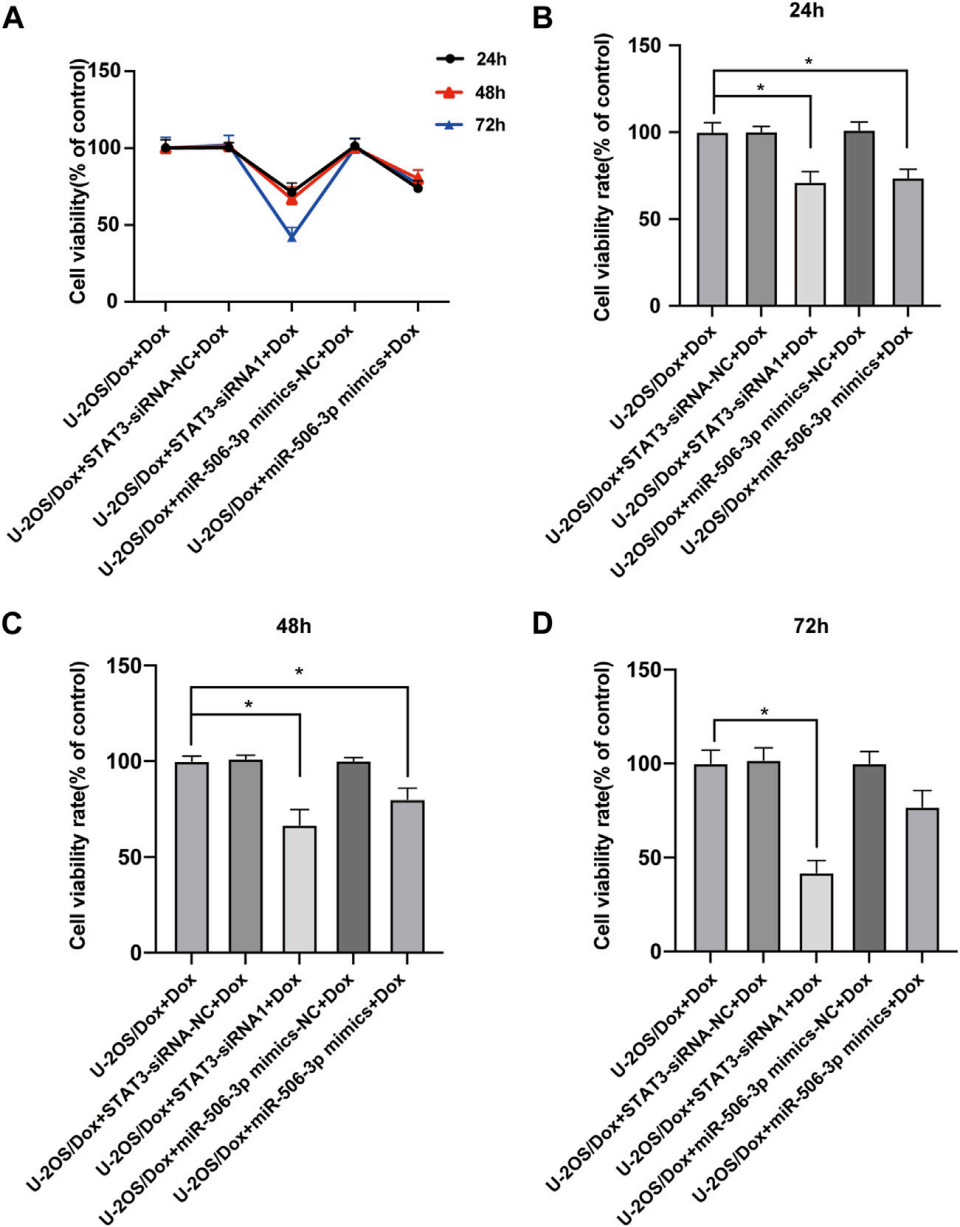
The proliferation abilities of drug-resistant osteosarcoma cells were both inhibited by miR-506-3p overexpression and silencing STAT3 **(A)** U-2OS/Dox cells were divided into 5 groups for different treatments, the cell proliferation abilities were detected using CCK8 assay after co-cultured with 29 μg/mL doxorubicin for 24 h, 48 h and 72 h **(B–D)** The cell proliferation activities of the overexpression of miR-506-3p group and the silencing of STAT3 group were both significantly reduced, compared with the U-2OS/Dox + Dox group. *: *p* < 0.05, **: *p* < 0.01.

**FIGURE 11:**
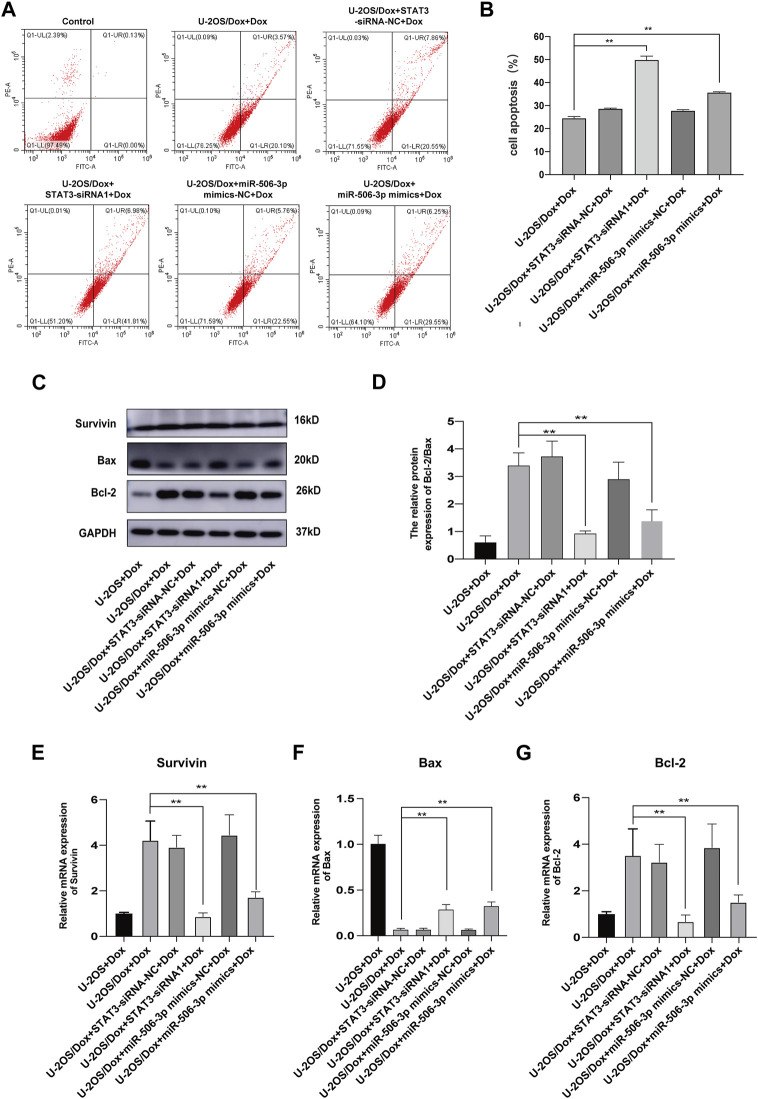
The apoptosis of drug-resistant osteosarcoma cells was both increased by miR-506-3p overexpression and silencing STAT3 **(A, B)** The apoptosis rate of each group was detected using Annexin V-FITC/PI double-stained apoptosis cassette. The apoptosis rates were both significantly higher in the overexpression of miR-506-3p group and the silencing of STAT3 group by flow cytometry. **(C)** The Western blot protein bands of Survivin, Bax and Bcl-2. **(D)** The relative protein expression ratio of Bcl-2/Bax was significantly reduced in the overexpression of miR-506-3p group and the silencing STAT3 group by Western blot. The mRNA expressions of the apoptotic proteins Survivin **(E)** and Bcl-2 **(G)** were both inhibited by overexpression of miR-506-3p and silencing of STAT3 through RT-qPCR, and the mRNA expression level of Bax **(F)** was significantly increased. **: *p* < 0.01.

After the U-2OS/Dox cells in each group were co-cultured with 29 μg/mL doxorubicin for 24 h, 48 h and 72 h, through CCK8 test, the cell proliferation activities in the overexpression miR-506-3p group and the STAT3 silencing group were significantly reduced compared with the control group (Figure 10, *p* < 0.05). Flow cytometry results showed that the apoptosis rate was significantly higher in the miR-506-3p overexpression group and the of STAT3 silencing group (Figures 11A, B, *p* < 0.01). The expressions of anti-apoptotic proteins Survivin and B-cell lymphoma-2 (Bcl-2) were detected by Western blot and RT-qPCR, and the results showed that the expressions of Survivin (Figures 11C, E) and Bcl-2 (Figures 11C, G) were significantly reduced in the miR-506-3p overexpression group and the STAT3 silencing group. The protein (Figure 11C) and mRNA (Figure 11F) expression levels of pro-apoptotic protein Bcl-2 associated X (Bax) were significantly increased. The relative protein expression ratio of Bcl-2/Bax was significantly reduced in the overexpression of miR-506-3p group and the silencing STAT3 group (Figure 11D, *p* < 0.01). The results above indicated that overexpression of miR-506-3p inhibited U-2OS/Dox cell proliferation and promoted U-2OS/Dox cell apoptosis, consistent with the effects of silencing STAT3.

### 2.6 Effects of miR-506-3p overexpression and STAT3 silencing on drug resistance pathway proteins in U-2OS/Dox cells

**FIGURE 12:**
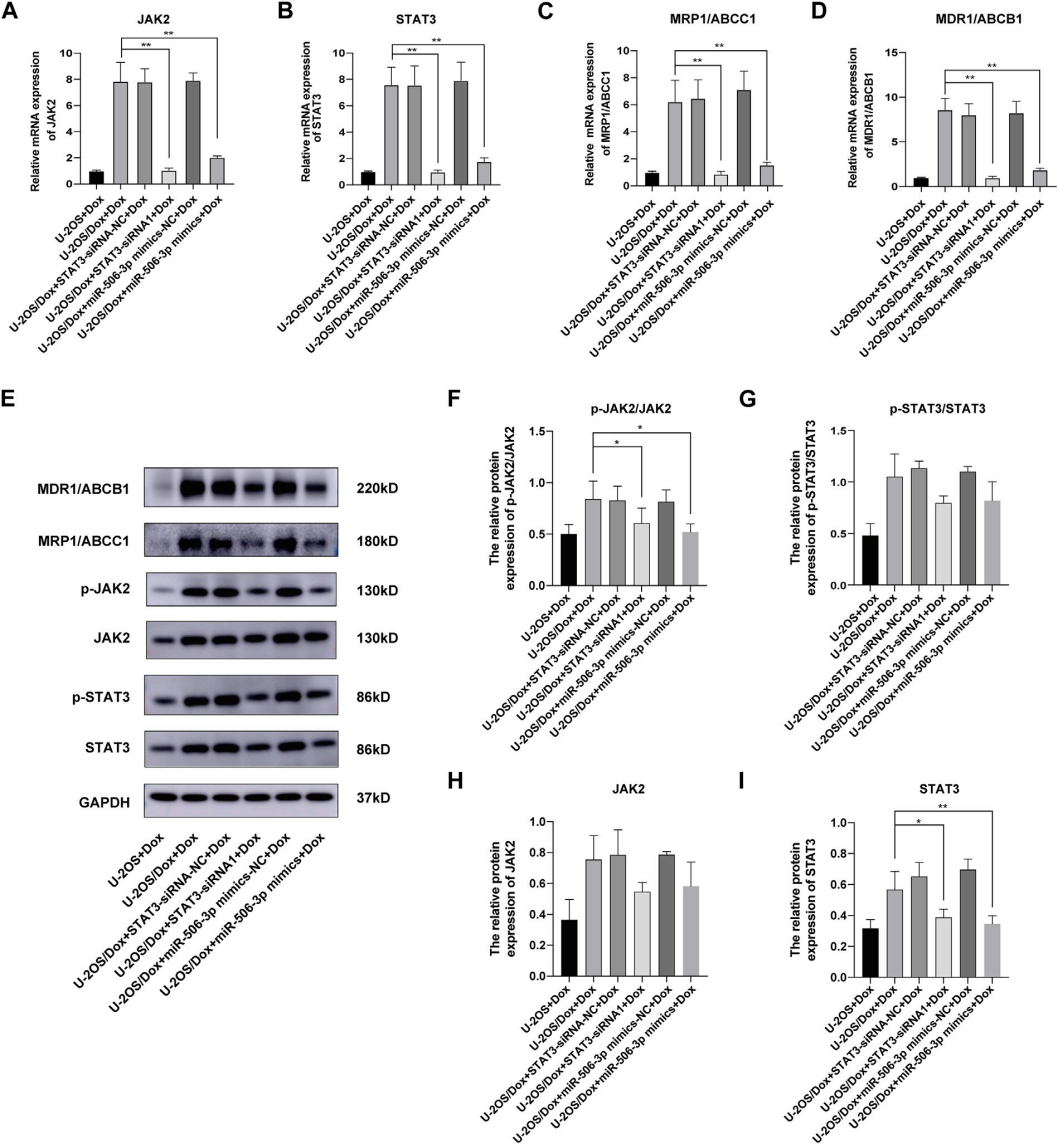
Overexpression of miR-506-3p and silencing STAT3 could both inhibit the expression of drug-resistant proteins in drug-resistant osteosarcoma cells by targeting the JAK2-STAT3 signaling pathway The mRNA expressions of JAK2 **(A)**, STAT3 **(B)**, MRP1/ABCC1 **(C)** and MDR1/ABCB1 **(D)** were both inhibited by miR-506-3p overexpression and silencing STAT3, compared with the U-2OS/Dox + Dox group. **(E)** The Western blot protein bands of STAT3, p-STAT3, JAK2, p-JAK2, MRP1/ABCC1 and MDR1/ABCB1. Densitometry analysis for p-JAK2/JAK2 **(F)**, p-STAT3/STAT3 **(G)**, JAK2 **(H)** and STAT3 **(I)**, showed that protein expression levels of p-JAK2/JAK2 and STAT3 were significantly reduced. *: *p* < 0.05, **: *p* < 0.01.

After doxorubicin was added to each group (U-2OS group, U-2OS/Dox group, U-2OS/Dox + STAT3-siRNA-NC group, U-2OS/Dox + STAT3-siRNA1 group, U-2OS/Dox + miR-506-3p mimics-NC group and U-2OS/Dox + miR-506-3p mimics group), compared with the U-2OS/Dox + Dox group, the mRNA expression levels of JAK2, STAT3, MRP1/ABCC1 and MDR1/ABCB1 were all remarkably inhibited in the miR-506-3p overexpression group and the STAT3 silencing group (Figures 12A–D, *p* < 0.01). However, at the protein expression level, overexpression of miR-506-3p and silencing of STAT3 significantly reduced the expression levels of p-JAK2/JAK2 (Figures 12E, F, *p* < 0.05) and STAT3 protein (Figures 12E, I, *p* < 0.05), which also reduced the expression levels of p-STAT3/STAT3 (Figures 12E, G) and JAK2 protein (Figures 12E, H), but there was no statistically significant difference. The results suggested that miR-506-3p overexpression inhibited the JAK2/STAT3 signaling pathway, thereby reversed the chemoresistance of U-2OS/Dox cells to doxorubicin, consistent with the results of STAT3 silencing.

## 3 Discussion

Currently, chemotherapy had become one of the mainstream treatments for osteosarcoma. MAP, consisting of methotrexate, doxorubicin and cisplatin, was the most widely available chemotherapy regimen for osteosarcoma (Hu et al., 2022). Unfortunately, some osteosarcoma patients showed poor chemosensitivity to these antitumor agents, and they were prone to chemoresistance during treatment, even with rising dose, shortening the period of chemotherapy or changing interval with other anticancer drugs. These patients still could not avoid recurrence and metastasis (Xiao et al., 2018). Doxorubicin, as the first-line drug for osteosarcoma, could induce osteosarcoma cell apoptosis and activated host immune response against tumor-specific antigens (Hattinger et al., 2015). However, due to serious drug resistance issues, osteosarcoma patients were greatly limited in their choice of regimens.

STATs were a family of proteins that existed in the cytoplasm and could be translocated into the nucleus to bind to DNA upon activation, which had the dual functions of signal transduction and transcriptional regulation. STAT3, an important member of the family of STATs, had been recognized as an oncogene that was closely related to tumor development (Barton, 2006). Studies had shown that STAT3 was highly expressed and continuously activated in osteosarcoma, which was involved in various pathological processes such as cell transformation, proliferation, tumor formation, invasion, migration, immune escape and drug resistance (Salas et al., 2014; Zhang et al., 2017). Inhibition of STAT3 activity had been shown to improve the chemosensitivity of osteosarcoma cells (Wang et al., 2011; Liu et al., 2021). We found that silencing STAT3 inhibited the cells proliferation, migration, and invasion ability of U-2OS/Dox cells and promoted cells apoptosis.

Studies had demonstrated that there were a large number of differentially expressed miRNAs in osteosarcoma tissues and cell lines. Some of these miRNAs were related to the development of osteosarcoma, while others were involved in the regulation of drug resistance in osteosarcoma (Tang et al., 2021). High expression of miR-124 in tumor tissues was a favorable prognostic marker for osteosarcoma (Wang et al., 2016; Cong et al., 2018). Three other studies have confirmed that miR-124 could inhibit the protein expression of STAT3 by directly targeting the 3′-UTR that binds STAT3, thereby suppress the invasive and migratory ability of tumor cells and help to overcome chemoresistance (Hu et al., 2016; Liu et al., 2019; Qi et al., 2019). We found that the seed sequence of hsa-miR-506-3p converged with that of hsa-miR-124 by pre-bioinformatics analysis, and the predicted that the binding site of miR-506-3p to the 3′-UTR of target gene STAT3 was broadly conserved with miR-124. Therefore, we predict that miR-506-3p is also able to directly target STAT3. MiR-506-3p played an important role in malignant tumor proliferation, apoptosis, migration, invasion, and epithelial-mesenchymal transition (Wang et al., 2019; Li et al., 2020; Liu et al., 2020). Moreover, it was closely related to tumor resistance. MiR-506-3p mainly played an antitumor role, and could also increase the sensitivity of osteosarcoma to cisplatin (Dong and Qu, 2020). However, no studies have been reported on the role of miR-506-3p in the development of osteosarcoma and reversal of doxorubicin resistance by targeting STAT3. Our study found that miR-506-3p was significantly inhibited in U-2OS/Dox cells and further acquired the key target genes regulated by miR-506-3p, including STAT3. To fully understand the targeting relationship between miR-506-3p and STAT3, we performed dual luciferase reporter gene assay experiments, and demonstrated that there was a complementary binding sequence with miR-506-3p in the base sequence of STAT3 3′-UTR. In addition, the experimental results showed that overexpression of miR-506-3p reduced the STAT3 protein expression level in doxorubicin-resistant osteosarcoma cells U-2OS/Dox, indicating that miR-506-3p could inhibit the expression of STAT3 in osteosarcoma cells.

Tumor invasion and metastasis are complex processes regulated by multiple factors and multiple steps, involving tumor cells swimming out of the primary tumor, breaking through the basement membrane, passing through the cell stroma, entering blood vessels or lymphatic vessels, migrating to distant sites, finally colonizing and growing. The interactions of a variety of genes together formed a complex molecular biology network, which was the main biological feature of malignant tumors different from benign tumors (Hu et al., 2015; Chen et al., 2019). The effective control of proliferation, migration and invasion can inhibit the occurrence and development of tumors to a certain extent. It had been demonstrated that many miRNAs played a critical regulatory role in invasion and migration of osteosarcoma cells, including miR-506-3p (Jiashi et al., 2018). Our study also showed that overexpression of miR-506-3p inhibited the proliferation, migration and invasion of U-2OS/Dox cells, confirming that miR-506-3p plays an antitumor role in osteosarcoma.

Apoptosis was a tightly regulated cellular signaling process that occured via an extrinsic pathway of cell membrane death receptors and/or an intrinsic pathway dependent on mitochondria (Lossi, 2022). The Bcl-2 family played a key regulatory role in the extrinsic pathway of apoptosis, and consisted of pro-apoptotic proteins (such as Bax and Bak) and anti-apoptotic proteins (such as Bcl-2 and Bcl-xl). When the balance of anti-apoptotic and pro-apoptotic proteins in the Bcl-2 family was disrupted, it led to dysregulation of apoptosis (Wong, 2011). This defective apoptotic mechanism promoted the continued proliferation, angiogenesis and metastasis of tumor cells and was one of the major causes of carcinogenesis. Moreover, it had been demonstrated that defective tumor-induced apoptosis significantly raised the threshold of tumor cell death, thereby nullifying the cytotoxic effects of conventional chemotherapy and radiotherapy and mediating chemoresistance (Schimmer, 2004). We found that the expression of Bcl-2 was higher in drug-resistant osteosarcoma cells than in drug-sensitive osteosarcoma cells after treated with doxorubicin, whereas the expression of pro-apoptotic proteins was the opposite, leading to defective apoptosis in drug-resistant osteosarcoma cells, inhibiting apoptosis and mediating doxorubicin chemoresistance. However, compared with drug-resistant osteosarcoma cells, overexpression of miR-506-3p or silencing of STAT3 decreased the expression of Bcl-2 and increased the expression of Bax, which in turn led to a decrease in the Bcl-2/Bax ratio. This result reversed the dysregulation of apoptosis in drug-resistant osteosarcoma cells and promoted apoptosis, thereby improving the sensitivity of drug-resistant osteosarcoma cells to doxorubicin. Moreover, it had been found that Survivin expression was elevated in tumor tissues of chemotherapy-resistant osteosarcoma patients, and that continued chemotherapy could mediate the enrichment of high Survivin-expressing osteosarcoma cells, which ultimately lead to the development of multidrug resistance in the clinic (Wei et al., 2021). As an important downstream effector, downregulation of Survivin expression significantly reduces the incidence of chemoresistance development in osteosarcoma (Tsai et al., 2014). Our study also confirmed that Survivin was highly expressed in osteosarcoma-resistant cells, and overexpression of miR-506-3p or silencing of STAT3 was able to inhibit Survivin expression, thereby enhancing the sensitivity of drug-resistant osteosarcoma cells to doxorubicin.

The higher degree of drug accumulation in tumor cells, the stronger ability to kill tumor cells. The efficacy of doxorubicin, a substrate of p-glycoprotein (P-gP), is influenced by the active efflux of the drug mediated by the ATP-binding cassette (ABC) transporter protein. The efflux effect led to a decrease in intracellular doxorubicin concentration and the development of acquired resistance, which given rise to recurrence or deterioration in nearly one-third of osteosarcoma patients (Kathawala et al., 2015). It had been demonstrated that inhibition of MDR1/ABCB1 or MRP1/ABCC1 in the ABC transporter protein family could reverse doxorubicin resistance in tumor cells including osteosarcoma (Tsang et al., 2003; Sampson et al., 2019; Gerardo-Ramirez et al., 2022; Packeiser et al., 2023). The results of the present study showed that the expression levels of both MDR1/ABCB1 and MRP1/ABCC1 in drug-resistant osteosarcoma cells line U-2OS/Dox were significantly higher than those in drug-sensitive osteosarcoma cells line U-2OS. The enhanced action of these membrane efflux pumps led to a decrease in the concentration of antitumor drugs in osteosarcoma cells, thus, acquired resistance occurred (Gonzalez-Fernandez et al., 2017). However, decreasing the expression levels of MDR1/ABCB1 and MRP1/ABCC1 in U-2OS/Dox increased the chemosensitivity of drug-resistant osteosarcoma cells to doxorubicin.

JAK2 was a tyrosine kinase that existed under the cell membrane, and it could bind to various cytokine receptors to activate transcription factors such as STAT3 (Huang et al., 2022), an intranuclear transcription factor that promotes cell proliferation, differentiation, apoptosis, and immune response (Ma et al., 2020). Under normal physiological conditions, the JAK2/STAT3 signaling pathway was involved in many important biological processes, such as embryonic development, immune response, cell proliferation and differentiation. However, under certain pathological conditions, the JAK2/STAT3 signaling pathway was also abnormally activated, cytokines bind to their corresponding receptors, leading to recruitment of related JAKs, further change to p-JAK, JAK activation leads to formation of docking sites for STAT, STATs are phosphorylated to p-STAT by tyrosine, STATs dissociate from the receptor to form dimers, STAT dimers enter the nucleus, bind to DNA, and regulate transcription, the JAK/STAT signaling pathway is activated (Hu et al., 2021; Wang et al., 2022). Our study found that overexpression of miR-506-3p and silencing STAT3 inhibited the mRNA expression levels of JAK2 and STAT3 genes. However, at the protein expression level, overexpression of miR-506-3p and silencing of STAT3 expression had a significant inhibitory effect on the expression of p-JAK2/JAK2 and STAT3 total proteins, and there were no statistically significant differences on the expression of p-STAT3/STAT3 and JAK2 total proteins. It suggested that miR-506-3p might be able to reverse chemoresistance to doxorubicin in drug-resistant osteosarcoma cells by inhibiting the phosphorylation of JAK2 and the expression of total STAT3 protein in drug-resistant osteosarcoma cells and inhibiting the over-activation of JAK2/STAT3 signaling pathway. Of course, the specific mechanism of miR-506-3p in the regulation of JAK2/STAT3 signaling pathway in Doxorubicin-resistant osteosarcoma patients needs to be further investigated.

## 4 Conclusion

In summary, the study demonstrated that miR-506-3p could inhibit JAK2/STAT3 signaling pathway by directly binding STAT3, thus reversed the malignant biological behaviors such as proliferation, migration and invasion of drug-resistant osteosarcoma cells, promoted cell apoptosis, as well as inhibited the expressions of drug-resistant proteins. Our study provided theoretical support for improving chemical sensitivity of drug-resistant osteosarcoma cells in an attempt to solve the problem of doxorubicin resistance in osteosarcoma treatment.

## 5 Materials and methods

### 5.1 Construction and cell culture of drug-resistant osteosarcoma cell line U-2OS/Dox

The human osteosarcoma cell line U-2OS was obtained from the Chinese Academy of Sciences (Shanghai, China). The doxorubicin-resistant cell line U-2OS/Dox was measured using an intermittent stepwise increase method (Serra et al., 1993). U-2OS cells in logarithmic phase were taken, and doxorubicin was used as the inducing drug, and drug resistance of U-2OS cells was induced by gradually increasing the drug concentration of doxorubicin (30 ng/mL, 100 ng/mL and 580 ng/mL), and each concentration was shocked for 9 times, and then after the cells grew stably in this concentration, the concentration of the drug was increased to continue the culture. Doxorubicin drug induction lasted 6–8 months until the cells were able to grow stably in 580 ng/mL doxorubicin. The IC_50_ of the resistant cell lines was detected and the RI was calculated. RI = [IC_50_ of the resistant cell line]/[IC_50_ of the parental cell line], and RI > 10 was considered that the resistant cell line met the requirements. Both cell lines were cultured in McCoys (GIBCO, Invitrogen, Carlsbad, CA, United States) supplemented with 10% fetal bovine serum (GIBCO, Invitrogen, Carlsbad, CA, United States) in a constant temperature incubator at 37°C with 5% CO_2_.

### 5.2 Bioinformatics predictions

TargetScan Human 8.0 (https://www.targetscan.org/vert_80 (McGeary et al., 2019) was used to predict biological targets of miR-506-3p by searching for the presence of conserved 8mer, 7mer, and 6mer sites that match the seed region of each miRNA. Tumor-miRNA-Pathway (http://bioinfo.life.hust.edu.cn/miR_path/index.html) (Ma et al., 2016), miRWalk (http://mirwalk.umm.uni-heidelberg.de/) (Sticht et al., 2018) and miRDB (https://mirdb.org/) (Chen and Wang, 2020) were used to predict the target genes of miR-506-3p. Then the intersection of three databases was taken. We also searched the expression profiles of miR-506-3p in different tumor tissues and their paracancerous tissues using the Tumor-miRNA-Pathway database. After that, GO and KEGG enrichment analysis of the screened miR-506-3p target genes was performed using the DAVID database (https://david.ncifcrf.gov/home.jsp) (Jiao et al., 2012). Finally, PPI networks were constructed, including physical and functional associations, for the selected miR-506-3p target genes using the STRING database (https://cn.string-db.org/) (Szklarczyk et al., 2021). We selected the default parameter of the system, a medium confidence of 0.400, to screen 30 interacting target genes. In addition, the hub target genes were explored using the cytoHubba plug-in and degree topology algorithm in Cytoscape (version 3.8.0) software.

### 5.3 Dual luciferase reporter gene assay

The 293T cells and target plasmids were prepared for transfection beforehand, when the cell density reached 50%–70%. Solution A was prepared by combining 10 μL DMEM, 0.16 μg of h-STAT3-3′-UTR target plasmid and 5 p.m. of hsa-miR-506-3p NC at room temperature. Solution B was prepared by combining 10 μL DMEM with 0.3 μL transfection reagent (Hanhen Bio-products LipoFiter™, the concentration of which is 0.8 mg/mL) and left at room temperature for 5 min. Solution A and solution B were thoroughly mixed and left for 20 min. After 6 h of transfection, fresh medium was changed, and the cells were collected after 48 h of transfection, and the luciferase activity was detected by fluorescence detector.

### 5.4 Cell transfection

U-2OS cells and U-2OS/Dox cells were used as transfection targets in logarithmic growth phase. Cells were inoculated in 6-well plates 24 h prior to transfection, and transfection was carried out when the cell confluence was about 50%, and the cell culture medium was changed 30 min prior to transfection, with reference to the instructions of the transfection reagent of lipofectamine 3000 (thermo, L3000001, United States). The cells were first respectively transfected into U-2OS/Dox cells with STAT3-siRNA1, siRNA2, siRNA3 and siRNA-NC (Ribobio, Guangzhou, China) at the final concentration of 50 nM, which were targeted to the STAT3 mRNA and screened for the best STAT3-siRNA. MiR-506-3p mimics and miR-506-3p mimics-NC (Hanheng, Shanghai, China) were transfected into U-2OS/Dox cells at a final concentration of 50 nM, and cells were harvested 48 h after transfection. The primers of STAT3-siRNA1, siRNA2, siRNA3, miR-506-3p mimics and miR-506-3p mimics-NC were 5′-GGC​GTC​CAG​TTC​ACT​ACT​A-3′, 5′-AGA​CCC​GTC​AAC​AAA​TTA​A-3′, 5′-CAT​CGA​GCA​GCT​GAC​TAC​A-3′, 5′-UAA​GGC​ACC​CUU​CUG​AGU​AGA-3′ and 5′-UCA​CAA​CCU​CCU​CCU​GAG​UAG​A-3′, respectively.

### 5.5 CCK8 assay

#### 5.5.1 CCK8 detection of U-2OS and U-2OS/Dox cell viability

U-2OS and U-2OS/Dox cells were inoculated into 96-well plates at 6×10^3^/well for 24 h. Increasing concentrations of doxorubicin (0, 0.5, 1, 2, 4, 8, 16, 32 μg/mL) were added to the plates and blank control wells without cytosol were set up, with three replicate wells for each treatment. After 24 h of incubation, 10 μL CCK8 reagent (Kemix, Beijing, China) was added to each well, and the absorbance at 450 nm was measured on an enzyme labeler after another 1.5 h of incubation (A). Cell viability (%) = [A (experiment)—A (blank)]/[A (control)—A (blank)] × 100%. The IC_50_ values of U-2OS and U-2OS/Dox cells were determined by plotting the concentration-effect histogram with the drug concentration as the horizontal coordinate and the cell survival rate as the vertical coordinate.

#### 5.5.2 CCK8 detection of U-2OS/Dox cell viability after transfection with miR-506-3p mimics

The cells were divided into five groups: blank control group (U-2OS/Dox group), negative control group (U-2OS/Dox + STAT3-siRNA-NC group, U-2OS/Dox + miR-506-3p mimics-NC group) and experimental group (U-2OS/Dox + STAT3-siRNA1 group, U-2OS/Dox + miR-506-3p mimics group). The group-treated cells in step 5.4 were inoculated into 96-well plates and pre-cultured for 24 h, 48 h and 72 h. Then 10 μL CCK8 reagents was added, and then the absorbance at 450 nm wavelength was measured on the enzyme labeling instrument after incubation for another 1.5 h. The absorbance at 450 nm wavelength was determined (A). Cell viability (%) = [A (experiment)—A (blank)]/[A (control)—A (blank)] × 100%. The difference in cell viability of U-2OS/Dox cells transfected with miR-506-3p mimics was analyzed and compared with that of U-2OS/Dox cells of the original drug-resistant strain.

### 5.6 RT-qPCR assay

The cells were divided into 6 groups: U-2OS group, blank control group (U-2OS/Dox group), negative control group (U-2OS/Dox + STAT3-siRNA-NC group, U-2OS/Dox + miR-506-3p mimics-NC group) and experimental group (U-2OS/Dox + STAT3-siRNA1 group, U-2OS/Dox + miR-506-3p mimics group).The cells in logarithmic growth phase were taken and the total RNA in the cells was extracted using Trizol reagent total RNA extraction kit (Vazyme, Nanjing, China). MiRNA and mRNA were reverse transcribed into cDNA according to the procedure of Reverse Transcription Kit (Takara, Japan), the reaction was terminated after incubation at 50°C for 15 min and at 75°C for 5 min, and then subjected to real-time fluorescence quantitative analysis. The mRNA fluorescence quantification was performed by dye method, and the final concentration of both forward primer and reverse primer was 50 nM. Predegeneration was 95°C for 5 min, and amplification was performed at 95°C for 10 s, 60°C for 10 s, and 72°C for 10 s, a total of 40 cycles. The solubility curves were plotted and analysed based on 2^−ΔΔct^ calculations. All genes and their corresponding specific primer sequences (5′-3′) were shown in Table 1.

**TABLE 1:** The primers of genes used for RT-qPCR.

Gene name	Direction	Sequence
STAT3	Forward	AGC​AGC​ACC​TTC​AGG​ATG​TC
	Reverse	GCA​TCT​TCT​GCC​TGG​TCA​CT
JAK2	Forward	TGG​AGG​GAA​CAT​CCA​CCT​CT
	Reverse	TCT​GCC​TCA​GAT​TTC​CCA​AGG
miR-506-3p	Forward	ACC​ACC​GTA​AGG​CAC​CCT​TCT
	Reverse	ATC​CAG​TGC​AGG​GTC​CGA​GG
MDR1/ABCB1	Forward	GCT​GTC​AAG​GAA​GCC​AAT​GC
	Reverse	GAG​GAT​CTT​GGG​GTT​GCG​AA
MRP1/ABCC1	Forward	TCC​CCT​GAA​CAT​TCT​CCC​CA
	Reverse	ATG​CTG​TCA​GGT​TCC​AGC​TC
Survivin	Forward	GAG​GCT​GGC​TTC​ATC​CAC​T
	Reverse	TGG​TTT​CCT​TTG​CAT​GGG​GT
Bax	Forward	CCC​CCG​AGA​GGT​CTT​TTT​CC
	Reverse	CTG​ATC​AGT​TCC​GGC​ACC​TT
Bcl-2	Forward	TGG​TGG​AGG​AGC​TCT​TCA​GG
	Reverse	CTC​TCC​ACA​CAC​ATG​ACC​CC
GAPDH	Forward	ATT​CCA​CCC​ATG​GCA​AAT​TCC
	Reverse	GAC​TCC​ACG​ACG​TAC​TCA​GC
U6 (by stem-loop)	Forward	AGA​GAA​GAT​TAG​CAT​GGC​CCC​TG
	Reverse	CAGTGCAGGGTCCGAGGT

### 5.7 Western blot assay

The cells were centrifuged at 2500 rpm for 5 min, then protein cleavaged with strong RIPA lysis buffer (CWBIO, Jiangsu, China), and centrifuged at 13,000 rpm for 6 min at 4°C to collect the total proteins, and the protein concentration was measured by BCA kit. After denaturation at 98°C for 10 min, the proteins were separated by 10% SDS-PAGE and transferred onto a PVDF membrane. The membrane was incubated with primary antibody at 4°C overnight, rinsed, and then incubated with secondary antibody at room temperature for 2 h. Finally, enhanced chemiluminescence (ECL) reaction was performed and visualized, and the images were processed using ImageJ software to calculate the relative protein expression levels. Primary antibodies were anti-STAT3 (1:1000, #30835, CST, Danvers, MA, United States), anti-p-STAT3 (1:1000, #94994, CST), anti-JAK2 (1:1000, YT2426, ImmunoWay, United States), anti-p-JAK2 (1:1000, YP0306, ImmunoWay), anti-MDR1/ABCB1 (1:1000, #13342, CST), anti-MRP1/ABCC1 (1:1000, #14685, CST), anti-Survivin (1:1000, #2808, CST), anti-Bax (1:1000, #2772, CST), anti-Bcl-2 (1:1000, #3498, CST), and anti-β-actin (1:1000, #8457, CST), anti-GAPDH (1:10000, AP0063, Bioworld, United States). Secondary antibodies were HPR-conjugated goat anti-mouse (1:10000, abs20039, absin, Shanghai, China), goat anti-rabbit (1:10000, abs20040, absin), and goat anti-rabbit (1:10000, E030120-01, Earthox, United States).

### 5.8 Cell mobility scratch assay

The cells of each group were inoculated in 6-well plates at a density of 2×10^5^/well, and transfection was carried out when the cell density reached 50%. After the cells were spread all over the bottom of the plate, the cells were gently scratched vertically with the tip of a 200 μL pipette and rinsed gently with PBS for 2 times, and then cultured in low-serum medium (containing 2% FBS) for 24 h. The cellular healing images of the scratches were observed and photographed under a microscope at the moment of scratches (0 h), 24 h and 48 h, and the widths of the scratches were measured to calculate the cell migration force. Cell migration force = [scratch width (0 h)—scratch width (24 h or 48 h)]/scratch width (0 h) × 100%.

### 5.9 Transwell assay for cell invasiveness

Two hundreds microliter of cell suspension for each group (5×10^4^ cells) were added to the Transwell coated with Matrigel matrix gel, and 600 μL complete culture medium containing 10% FBS was added to the lower chamber. After incubation for 24 h, the non-migrated cells were wiped off from the membrane of the upper chamber, and the membrane of the lower chamber was fixed with 4% paraformaldehyde for 10 min. After drying naturally, the membrane was stained with 0.5% crystalline violet solution for 15 min at room temperature, and then observed under an inverted microscope. Three 100 × fields of view were randomly taken for photographing, and the total number of cells in the five different fields of view (top, bottom, left, right and center) were counted and averaged to calculate the cell migration rate.

### 5.10 Flow cytometry

Cells were digested with EDTA-free trypsin, and the density of each cell suspension was adjusted to 2×10^5^ cells/mL. One millilitre of cell suspension was added into a sterile centrifuge tube, and the cells were centrifuged at 2000 rpm for 5 min at room temperature to collect the cells, and the supernate was discarded. The cells were washed twice with pre-cooled PBS (4°C), and 300 μL 1× binding buffer was added to resuspend the cells. Cells were washed twice with pre-cooled PBS (4°C), resuspended by adding 300 μL 1× binding buffer. According to the instructions of the Annexin V-FITC/PI Dual Staining Apoptosis Detection Kit (BD Biosciences, 556547, United States), incubated for 15 min at room temperature with 5 μL Annexin V-FITC, and then 5 μL PI was added for 5 min prior to the start-up of the detection. The apoptosis rate of each group was detected by flow cytometry, and the total apoptosis rate (%) = [early apoptosis rate (Q4 quadrant percentage)] + [late apoptosis rate (Q2 quadrant percentage)].

### 5.11 Statistical analysis

Data were obtained from at least three independent replicated experiments and the results were expressed as mean ± standard deviation (‾x ± s). Data were analyzed and processed using IBM SPSS 24.0 and GraphPad Prism 9.0.0 software. Comparisons between multiple groups were analyzed by one-way ANOVA or Kruskal-Walis H Test, and comparisons between two groups were made by *t*-Test or Mann-Whitney *U* Test, with *p* < 0.05 indicating that the differences were statistically significant.

## Figures and Tables

**Table:** 

**STAT3**	signal transducer and activator of transcription 3
**JAK2**	janus kinase 2
**miRNA**	microRNA
**U-2OS/Dox**	doxorubicin-resistant U-2OS cells
**mRNA**	messenger ribonucleic acid
**p-JAK2**	phospho-JAK2
**p-STAT3**	phospho-STAT3
**P-gP**	P-glycoprotein
**MDR1**	multi-drug resistance protein 1
**ABCB1**	ATP-binding cassette subfamily B member 1
**MRP1**	multi-drug resistant associate protein 1
**ABCC1**	ATP-binding cassette subfamily C member 1
**Bax**	Bcl-2 associated X
**Bcl-2**	B-cell lymphoma-2
**RT-qPCR**	real time quantitative polymerase chain reaction
**CCK8**	cell counting kit-8
**3′-UTR**	3′-untranslated region
**IC**_**50**_	half inhibitory concentration
**RI**	resistance index
**NC**	negative control
**TCGA**	the cancer genome atlas
**GO**	Gene Ontology
**KEGG**	Kyoto Encyclopedia of Genes and Genomes
**BP**	biological processes
**MF**	molecular function
**CC**	cellular component
**FoxO**	Forkhead Box O
**EGFR-TKI**	epidermal growth factor receptor-tyrosine kinase inhibitor
**ErbB**	receptor protein-tyrosine kinase
**PPI**	protein-protein interaction
**AKT2**	RAC-beta serine/threonine-protein kinase
**BCL2L11**	B-cell lymphoma 2-like 11
**MEF2A**	myocyte enhancer factor 2A
**SOD2**	Recombinant ruperoxide dismutase 2
**FXN**	Frataxin
**HDAC4**	Histone deacetylase 4

## Data Availability

The original contributions presented in the study are included in the article/Supplementary Material, further inquiries can be directed to the corresponding authors.
